# Association of Dietary Intake with Chronic Disease and Human Health

**DOI:** 10.3390/nu17030446

**Published:** 2025-01-26

**Authors:** Domenico Tafuri, Francesca Latino

**Affiliations:** 1 Department of Medical, Motor and Wellness Sciences, University of Naples “Parthenope”, via Medina 40, 80133 Naples, Italy; domenico.tafuri@uniparthenope.it; 2 Department of Education and Sport Sciences, Pegaso University, 80100 Naples, Italy

## 1. Introduction

Eating habits are among the major determinants of health, and in most countries in the world part of the population suffers from one or more problems related to malnutrition, by default (due to an acute or chronic lack of micronutrients) or by excess (energy, sugars, and fats, and deficiencies in important micronutrients) [1]. Extensive scientific literature has long confirmed that diet has a strong impact on the health status of a population. The most relevant factors and diseases related to poor nutrition are obesity, diabetes, cardiovascular diseases, tumors, non-alcoholic fatty liver disease, chronic intestinal diseases, neuropsychiatric diseases, and neurodegenerative diseases [2,3].

The spread of unhealthy eating habits has caused an increase in overweight rates for almost 60 percent of the world’s population, a third of which suffer from obesity. These problems are associated with diseases such as cardiovascular disease, type 2 diabetes, and some forms of cancer. Major social and economic changes, such as trends towards globalization and urbanization, challenge the ability to eat healthy diets [4]. Products with low nutritional value challenge food cultures and traditions. Sedentary lifestyles and a lack of physical activity increase progressively with age and affect more than one in three adults, compounding the problem of unhealthy eating patterns [5]. According to the Atlas of Heart Disease and Stroke, recently published by the World Health Organization (WHO) [6], the combination of an unhealthy diet and a sedentary lifestyle is the second most responsible cause of the 17 million deaths from cardiovascular and brain diseases, preceded only by smoking, and of cancer. A varied and balanced diet reduces the risk of cancer [7].

Healthy eating and good nutrition are biological acts necessary for meeting the energy needs of individuals and are fundamental for their health [8]. The busyness that very often characterizes our lives compels the selection of convenience foods and eating too quickly, which challenge healthy eating patterns. Healthy diets are a very powerful tool in the prevention of pathological conditions that can compromise quality of life and even life expectancy. It is, at this point, conceivable how non-communicable diseases represent a threat to the goals of the 2030 Agenda [9], in terms of both affecting sustainable development and their high social costs. In fact, the global burden of non-communicable diseases, with the threat they pose, constitutes a serious public health problem, hampering social and economic development worldwide.

## 2. Nutrition as a Key Modifiable Factor for the Prevention of Chronic Disease and the Promotion of Human Health

### 2.1. Prevention of Chronic Disease by Means of Diet and Lifestyle Changes

Several studies have confirmed the significant role of foods in the development of certain forms of cancer and, conversely, the ability of other foods to prevent their formation [10,11]. According to the American Institute for Cancer Research (AICR), more than 30% of cancers are directly attributable to a poor diet, understood in both quantitative and qualitative terms. In this Special Issue, Wang et al. [12] explore the potential role of selenium in the developmental process of esophageal cancer. To explore the relationship between selenium-related factors and esophageal cancer through bioinformatic analysis, a case–control study was conducted to verify the results. The findings showed lower glutathione peroxidase 3 (GPx3) expression in tumor tissues compared to normal tissues. The findings did not establish a direct association between selenium itself and esophageal squamous cancer; however, the significant difference observed between GPx3 and core protein fatty acid binding protein 1 (FABP1) in the two groups suggested the potential significance of selenium in esophageal cancer prevention.

In addition, particular behaviors and habits can significantly affect individual cardiovascular risk: often the simple correction of these situations substantially modifies the incidence of coronary heart disease and therefore represents an important measure in cardiovascular prevention [13,14]. These aspects are highlighted by Sikand and Severson [15], who elucidate that suboptimal dietary quality has eclipsed all other mortality risk determinants, culminating in 11 million fatalities and constituting half of global cardiovascular disease mortality. In their investigation, they assert that adults are encouraged to adhere to a cardioprotective diet that prioritizes plant-based alimentary sources, including vegetables, fruits, legumes, nuts, and whole grains, alongside lean protein sources and fish. They are advised to curtail the consumption of foods replete in saturated fats and dietary cholesterol while minimizing sodium intake. Furthermore, the avoidance of trans-fats, processed meats, refined carbohydrates, and sugar-sweetened consumables is recommended. The incorporation of dietary adjuncts, such as viscous fiber, plant sterols/stanols, and long-chain omega-3 fatty acids, is also advocated. They concluded that the implementation of medical nutrition therapy resulted in enhancements in lipid profiles, weight management, HbA1c levels, and hypertension, alongside fiscal savings. Empirical studies indicate that nutrition education and engagement in physical activity can equip students with the ability to discern the influence of a healthful lifestyle on psychosomatic well-being. Additionally, insufficient physical activity coupled with a substandard diet can adversely affect children’s weight status, metabolic function, and cardiovascular health. In light of the immediate and prolonged ramifications of children’s health behaviors, there exists an urgent imperative to facilitate the adoption and sustenance of healthy lifestyles among children. Consequently, the enhancement of children’s physical activity and nutritional practices are recognized as two paramount public health initiatives.

Chen et al. [16] conducted an investigation into the impact of consistent nutritional consultations on the mitigation of risk factors pertinent to cardiovascular diseases, which include body mass index, body composition, blood pressure, lipid profiles, glucose-related biomarkers, and inflammatory mediators. The data were amassed from a sample of participants (n = 129) who underwent eight dietary consultations, subsequently categorized into two distinct groups based on the frequency of consultations: an irregular group (experiencing non-uniform consultation intervals; n = 39) and a regular group (who received consultations every three weeks; n = 90). In comparison to the irregular group, the regular group exhibited more pronounced declines in cardiovascular disease risk factors, encompassing body mass index, body fat percentage, triglyceride levels, total cholesterol, low-density lipoprotein cholesterol, and insulin concentrations. Furthermore, participants with a body mass index of ≥27 kg/m^2^ demonstrated significantly noteworthy enhancements in cardiovascular risk parameters, including body weight, body mass index, visceral fat mass, and levels of triglycerides, total cholesterol, low-density lipoprotein cholesterol, glycated hemoglobin, and insulin. The authors concluded that there exists substantial evidence supporting the efficacy of regular nutritional consultations for adults exhibiting cardiovascular disease risk factors, particularly among those classified as obese. Similarly, Capra and colleagues [17] underscore the significance of nutritional intervention as a preliminary therapeutic approach for individuals exhibiting elevated cardiovascular risk, with particular emphasis on its critical relevance for pediatric and adolescent populations. They scrutinize the predominant dietary patterns prevalent among children and adolescents, aiming to assess their impact on cardiovascular risk factors and the prevention of cardiovascular diseases. Their findings indicated that the Mediterranean diet, DASH diet, Nordic diet, and certain plant-based diets appear to be the most promising dietary frameworks concerning cardiovascular health during developmental stages. The analysis of the role of nutrition in the management of diabetes was another topic of investigation in this Special Issue. The fundamental role of diet in the management of diabetes and its self-control is undisputed; it is for all intents and purposes a therapy. Gal et al. [18] postulate that dietary habits and consumption behaviors are integral to both the prevention and management of type 2 diabetes mellitus (T2DM). Willey and associates [19] investigated dietary habits among a variety of ethnic, cultural, and regional groups. The researchers posited that individuals exhibiting a shared clinical phenotype (age, body mass index, duration since T2DM diagnosis, and insufficient glycemic regulation) would reveal analogous high-calorie “western” dietary patterns and suboptimal dietary quality, notwithstanding the variations in geographic locales and cultural backgrounds. Dietary information was obtained from a cohort of 611 adults across Argentina, Germany, Poland, Serbia, Slovakia, Slovenia, Spain, Turkey, and the USA through three 24 h dietary recalls. The authors inferred that the dietary pattern data indicate the persistence of influences from more traditional region-specific diets. Nevertheless, the overall quality of the diet was found to be inadequate, which may exacerbate insufficient glycemic control, potentially as a consequence of the excessive consumption of high-calorie, nutrient-poor foods, likely linked to the global transitions in the food supply that are currently underway. Finally, within this Special Issue, the crucial role of nutrition in the management of inflammatory bowel disease has been extensively discussed. Chronic inflammatory bowel diseases, characterized by a chronic recurrent inflammatory process affecting one or more intestinal segments, are strongly influenced by the intake of different foods. A proper diet cannot cure inflammatory bowel disease, but some foods can aggravate symptoms, while others can relieve them and promote healing. Palamenghi, Usta, Leone, and Graffigna [20] emphasize the importance of food intake in the management of inflammatory bowel disease (IBD) by offering valuable insights into the complex motivations that drive food choices among individuals with IBD. Based on their findings, food engagement had a significant impact on participants’ food preferences, with higher levels of food involvement associated with a propensity to seek out pleasurable food; however, individuals with high levels of health involvement reflected a better emotional state that resulted in a lower need for food. Sasson et al. [21] propose that dietary factors may exacerbate intestinal inflammation through the dysregulation of the immune response, modifications in intestinal permeability and the mucosal barrier, the promotion of microbial dysbiosis, and various other pathways. Consequently, modifications in dietary practices may be integrated into therapeutic approaches for inflammatory bowel diseases (IBDs).

### 2.2. Improving Nutrition Education at Schools: Challenges and Opportunities

Prevention is a fundamental activity for safeguarding health [22,23]. In this vein, schools, as institutions spread throughout the world, are the main place in which to exercise and strengthen food education processes as a preventive measure [24]. This could guide new generations to develop adequate sensitivity to issues of personal and collective well-being, sustainability, and the adoption of correct lifestyles. Concepts such as the value of food are important, through which one can rediscover a sense of belonging to a community in which food is always part of its history and place [25]. These values are also accompanied by multiple virtuous effects, such as the protection of production territories, supply chains, and food systems, the dissemination of the culture of responsible consumption, and the containment of food waste. The principle of prevention also makes health and welfare systems more sustainable, and a deep-rooted food culture helps entire populations make informed choices with a view to healthiness and sustainability [26]. These themes are the basis of the articles that represent this Special Issue. Moscatelli and colleagues [27] have highlighted how the years spent at university represent a critical period that can affect both quality of life and the eating habits of later adulthood, as well as, in the long term, the health of an individual. The aim of this study was to investigate the lifestyles of university students living far from home. Their study showed that it is possible to improve lifestyle through educational events aimed at making students aware of the health risks of unhealthy lifestyles.

Latino, Tafuri, Saraiello, and Tafuri [28] suggested that since youths spend the greater part of the day at school, this is considered an ideal setting for fostering active and healthy living. The authors investigated the connection between physical activity and academic achievement in normal-weight and overweight adolescents. One hundred students (aged 14–15) from a public high school in the south of Italy participated in either a 12-week classroom-based physical activity break program performed during science classes (60′/2 days per week) in which a nutritional educational program was carried out or in regular science lessons (60′/2 days per week). The conclusions of this research underpin the notion that classroom-based physical activity breaks are a successful approach for enhancing child students’ physical well-being.

### 2.3. The Potential of Nutrition to Promote Psychological and Behavioral Well-Being

Eating is one of the primary needs of all living beings, but it often represents much more than a tool to satisfy a physiological need of the body, and there are many psychological implications that are hidden behind every meal. Every meal, starting with the three main ones, is a very important social moment for human beings, who often like to eat in company [29].

Sometimes food becomes an outlet and something to cling to in fighting stress, anxiety or depression. It then happens that we eat too much or badly, favoring fatty or sweet foods, often very caloric, which also satisfy the need to fill a “void” from a chemical point of view. Other times, however, it may happen that we eat irregularly, ingesting everything possible and also giving in to real binge eating, which is very harmful in the long run for health and the maintenance of a normal weight.

In this perspective, Latino et al. [28] concluded that the association of a nutritional educational program with physical activity breaks is a successful approach for enhancing students’ psycho-physical well-being, as well as academic achievement.

Gheisary et al. [30] aim to provide a comprehensive list of associations between lifestyle traits and the general health and well-being status of individuals with arthritis in the Canadian population, using binary logistic regression analysis on data from the Canadian Community Health Survey, which includes 104,359 respondents. Their study highlights the need for personalized, holistic approaches that can include a combination of dietary interventions, sleep therapies, and smoking cessation for better prevention and lifestyle improvement.

Li and colleagues [31], on the other hand, underline in their study the importance of nutritional changes and, in particular, the intake of phytanic acid in the management of the symptoms and psychosocial implications that follow in patients with Refsum disease.

Shawon and colleagues [32] conducted an investigation into the sex-specific prevalence of psychological distress and maladaptive eating behaviors among adolescents across various nations and regions, with the aim of examining their potential correlations. The study utilized data obtained from the Global School-Based Health Survey (GSHS), encompassing 61 nations. Their results reveal a significant disparity in the prevalence of psychological distress and maladaptive dietary practices among distinct regions. Furthermore, adolescents who reported experiencing psychological distress were found to be at an increased likelihood of engaging in unhealthy eating behaviors.

### 2.4. Nutrition and Physical Activity in the Prevention and Treatment of Chronic Disease

In addition to what we eat, it is essential to always keep the body moving. The habit of physical activity, together with its frequency and intensity, contributes to reducing or increasing our energy needs [33].

With advancing age, body fat increases, especially visceral fat, water in the tissues decreases, and bone density as well as muscle mass are reduced, causing a drop in basic metabolism. This explains the importance of physical activity for the reduction in and control of body weight, now recommended for all healthy people (regardless of age). To preserve mental and physical well-being, the WHO [34] recommends that we do at least 150 min of moderate physical activity per week. It has been calculated, in fact, that 30 min a day is enough to prevent weight gain in a person who follows a varied and balanced diet. On the other hand, the commitment must increase (60–90 min a day) if the person has to lose weight or maintain the weight achieved by following a diet (the recommendation is all the more significant the greater the weight loss).

Sedentary behaviors, such as spending too much time in front of the television, are not only a harmful habit for young people, but also affect adults. In children and adolescents, multimodal interventions aim at the systematic reduction in sedentary lifestyles and the increase in habitual AF, which together with proper nutrition education could reduce the growth of overweight and obesity and ensure better levels of health in populations in the future (EU Action Plan on Childhood Obesity 2014–2020), as highlighted by the studies of Latino and colleagues in addition to Moscatelli and colleagues [27,28]. The interventions can be combined with a training approach, as the practice of AF activates in young maturation processes, promoting personal and social development [35].

Physical exercise is also an effective health intervention in the treatment of numerous chronic diseases and, in terms of reducing mortality, provides benefits similar to those obtainable from drugs in the secondary prevention of coronary heart disease, post-stroke rehabilitation, heart failure, and diabetes prevention; in less severe pathologies (back pain, osteoarthritis), it brings significant benefits to symptoms and quality of life [36].

According to this line of thought, Janiczak and colleagues [37] highlight the role of proper nutrition in the management of athlete recovery.

### 2.5. Environmental and Policy Approaches in the Prevention of Chronic Diseases: The Role of Governments and Health Systems

The role of the health system in the implementation of preventive programs should be to disseminate greater scientific knowledge of the role of nutrition in the public health and economic sustainability plans of governments [38,39,40,41,42,43]. Through primary prevention, an attempt is made to improve lifestyles in the population to reduce the burden of diseases. Lifestyle has a significant impact on health, as it acts on the main risk factors. Eating habits have a powerful influence on health, favoring it if body weight is kept within optimal limits.

The emergence of the importance of managing dietary habits in combating chronic disabling diseases has placed an urgent need on governments to develop sustainable and effective public intervention models [44,45,46,47]. So far, the effectiveness and convenience of certain types of preventive programs have been confirmed: those implemented in hospitals, workplaces, and those aimed at the elderly population. On the other hand, there is still insufficient evidence to understand which types of interventions are most effective in producing a significant and lasting increase in dietary changes, and which are most suitable for extending the benefits to large segments of the population.

This line of research is extensively explored worldwide. In their study, Downer and colleagues [48] critically assess contemporary initiatives aimed at integrating food and nutrition into the prevention, management, and therapeutic approaches for diet-related diseases within healthcare frameworks. The authors underscore the significance of interventions that are being implemented with increasing frequency in the United States and have been trialed to a certain degree in various other nations, including medically tailored meals, medically tailored grocery provisions, and produce prescription initiatives.

## 3. Conclusions

Overall, the fifteen studies enclosed within this Special Issue encompass both experimental and descriptive methodologies pertinent to design (for instance, correlational, survey, and developmental inquiries). It is significant to note that all of the research disclosed highly favorable outcomes regarding the association between nutrition, chronic diseases, and human health across diverse populations. These inquiries contribute to the existing body of knowledge in this domain. We aspire for these articles to be regarded as intellectually stimulating and academically engaging. Furthermore, we anticipate the emergence of additional studies employing innovative methodologies and advanced techniques to further explore this critical research domain and to advocate for healthier lifestyles.
